# The interconnected global emergencies of climate change, food security and health: a call to action by the Science for Africa Foundation

**DOI:** 10.12688/openresafrica.13566.1

**Published:** 2023-01-03

**Authors:** Thomas Kariuki, Judith Omumbo, Kabura Ciugu, Elizabeth Marincola

**Affiliations:** 1 Science for Africa Foundation, Nairobi, Kenya

**Keywords:** Africa priorities, climate emergency, research capacity, food security, health, Science for Africa Foundation, research strategy

## Abstract

The evidence is clear that climate change is the greatest challenge facing mankind today. Africa is disproportionately burdened by multiple direct and cascading impacts of the climate crisis. Global investments for climate change adaptation, however, have not prioritized Africa adequately and there is a significant knowledge gap in understanding the context and science of climate change and sustainable solutions for the continent’s adaptation. Solutions for adaptation and resilience are made complex by an urgent need for accelerated economic growth, rapid population expansion and urbanization, habitat and biodiversity loss and dwindling financing.  There are also challenges in matching policies, wavering commitments and actions with good science that focuses on sustainable lives, livelihoods and ecosystem preservation.

The solutions must come from where the impacts are felt. The Science for Africa Foundation supports African researchers and institutions to lead in the science that addresses African priority development areas and has set climate change as a strategic priority. This call to action, by the SFA Foundation, outlines key areas that its strategy addresses through programs that support African scientific excellence, leadership and the best of Africa’s research to understand the science of climate change and its impacts; collate and assess evidence for policy; grow high level technical capacity on the continent; and create innovative priority actions for Africa.

## Introduction

Relatively small increases in global average temperature, on the order of ^+^0.5° centigrade, are associated with global scale extreme events. According to the available record, the Earth’s land and ocean surfaces have been warming since the 1800’s [ Lindsey & Dahlman, 2022]. This heating has occurred in tandem with global industrialization, changes in farming methods, automated transportation and a continuing heavy reliance on fossil fuels. Persistence in burning fossil fuels has led to a man-made warming, primarily over land surfaces, that is unprecedented. The rate of global heating has doubled since 1981 such that climate change is now a global existential emergency ( Butler, 2018). Its degradation of the world’s dwindling land, air and water resources threatens the livelihoods of over 100 million people in Africa with increased impacts of extreme events, perennial food insecurity, mounting disease burden and conflict, as more people become displaced. Evidence shows that, by increasing greenhouse emissions, human actions have led to an unprecedented rise in the frequency and magnitude of extreme weather and climate events, including heat waves, wildfires, floods, drought and landslides ( Seneviratne *et al.*, 2012). The 2018 special report of the International Panel on Climate Change of the United Nations (IPCC) on climate change at 1.5°C ( IPCC, 2018) issued a stark warning and call to action by all countries with alarming messages for countries where heating at this level has already occurred and populations are disproportionately vulnerable.

According to the IPCC’s 6 ^th^ Assessment report ( IPCC, 2021), Africa is already experiencing increased mean temperatures and hot extremes above natural variability relative to 1850–1900; all of Africa’s land regions have recorded a more rapid increase in temperature compared with the global average that is predominantly due to human factors. This has been manifested in increased hot extremes and reduced cold extremes; increased marine heatwaves; sea levels that have risen and are more frequent, and intense rainfall events. These direct effects of warming are worsened by their compound and cascading impacts on Africa’s societies, including dependency, humanitarian crises, human conflict and the trapping of communities in perpetual cycles of poverty. The threat to Africa’s economies, health systems, ecosystems and agriculture requires the concerted effort of Africa’s scientists and governments to prioritize actions that will secure the continent’s adaptation and resilience to climate change.

The Science for Africa Foundation is a funding organization delivering transformative programs across Africa that are driven by African priorities for sustainable development. The SFA Foundation’s initiatives are managed through transparent governance and informed by the most relevant measurement outcomes. The SFA Foundation has identified climate change as a strategic priority. The SFA foundation strategy aims to develop programs that will support African scientific leadership in the understanding of the science of climate change in Africa, and its impacts across spatial and timescales; to further African expertise in climate science and add value to Africa’s climate modelling; to bring the best of Africa’s research together to collate and assess the evidence for regional governments; and to grow high level technical capacity on the continent so that global discussions on priority actions for Africa are informed by her climate change science and led by the continent’s scientists.

### The climate emergency

***A global crisis.*** Humanity has evolved over hundreds of thousands of years since emerging in Africa, having survived numerous hot-dry spells, the ice ages and other climate extremes. Scientific evidence that climate has had a significant role in human evolution has been available since at least the 1920s ( Dart, 1925), when scientists began to debate whether drier conditions led early human ancestors to begin walking on two feet as an adaptation to life on the African savannah. Recent research provided compelling evidence that climate has played a part in shaping humanity. Robust simulations of climate-induced habitat changes over the past two million years influenced early hominin distributions and migration in various locations including in Africa ( Timmermann *et al.*, 2022). Palaeobiological studies in southern and eastern Africa, in particular, have made a significant contribution to understanding the evolution of the spatial distributions of early hominin species due to the richness of archaeological, paleoanthropological and paleoenvironmental records. Scientists have modelled this information to reconstruct biogeographical biomes that show the habitats of Middle Stone Age hominins in eastern Africa ( Faith *et al.*, 2015; Timbrell *et al.*, 2022)

Climate drivers possibly contributed to the emergence of the modern-day human species around 300,000 years ago. Based on genetic studies of thousands of fossils and other archaeological evidence, mathematical models can reveal how changes in the Earth’s movement have influenced climate and human evolution. The combined results demonstrate how six species of humans, including the early Homo erectus and the modern Homo sapiens, lived in Africa. The results also suggest that our species evolved through the loss of liveable habitats during an unusually warm period, migrating away from and adapting to hotter, drier conditions.

Urbanization and complex societies have existed for no more than 10,000 years. During this period, known as the Holocene, Earth’s climate has been relatively stable. Modern humanity is different: now, global warming is presenting human societies with enormous unprecedented challenges. Confronting this complexity “…will be like conducting an open-ended experiment on billions of human guinea pigs” ( Harari, 2018) Even if human civilization eventually adapts to these new conditions, in the vast lands of Africa, people will continue to be dependent on stable, predictable weather seasons for food, nutrition and livelihoods and therefore, the prospect of irreversible climate change is the most threatening of all.

Humankind today faces the existential threat of ecological collapse that hardly registered on the political radar in the 1960s. Humans are destabilizing the global biosphere by taking more and more resources out of the environment while pumping back into it, enormous quantities of waste and poison, thereby changing the composition of soil, water and the atmosphere ( Harari, 2018). Some saw it coming and, fifty years ago, the System Dynamics group at the Massachusetts Institute of Technology issued a stark message for the world: continued economic and population growth would deplete Earth’s resources and lead to global economic collapse by 2070 ( Meadows *et al.*, 1972). This was a shocking forecast at the time, and many, including scientists, were sceptical. The suggestion that the foundations of industrialized civilization; unclean energy from coal and oil, manufacturing of steel and indiscriminate use of poisonous pesticides and fertilizers, might cause lasting damage, did not go down well. While research leaders recognized that industry pollutes air and water, they considered such damage reversible.

Today, human activity has modified 77% of the Earth's land (excluding Antarctica) and 87% of the oceans. Data show that 11 million metric tons of plastic waste enters the oceans every year and another 50 million tons of E-waste is produced annually ( The Pew Charitable Trusts & SystemIQ, 2020). This waste enters the marine food chain and ends up on our dinner tables via contaminated fish and seafood laced with pathogens and potential carcinogens.

***Have we reached the “tipping point?”*** The basic consensus that emerges from long-running scientific debate is that human activity, in particular the emission of greenhouse gases such as naturally occurring carbon dioxide, water vapor and methane, man-made hydrofluorocarbons (HFCs) and chlorofluorocarbons (CFCs) that trap the heat of the sun, are causing the Earth’s land and ocean surfaces to heat up at an alarming rate. Nobody knows exactly how much carbon dioxide we can continue to pump into the atmosphere without triggering an irreversible cataclysm. For this reason, scientists and policymakers have urged that we limit the rise in global warming to no more than 1.5°C ( IPCC, 2018). The best scientific estimates indicate that unless we dramatically cut the emission of greenhouse gases in the next 20 years, average global temperatures will increase by more than 2 degrees Celsius; we have already experienced the greatest rise in temperatures in history in 2020/22.

A rapid attribution study by climate scientists in India, posted on the World Weather Attribution Website ^1^, reported a 30-fold increase in the probability of heat waves in India that can be attributed to climate change ( Zachariah *et al.*, 2021). On 15 May 2022, India Meteorological Department observations from numerous stations reported temperatures of between 45°C (113°F) and 50°C (122 °F). This followed a heatwave at the end of April and early May, during which temperatures reached 43–46 °C. The heat was prolonged and widespread and coupled with below-average rainfall, impacted hundreds of millions of people in one of the most densely populated parts of the world. Meteorological and hydrological departments in India and Pakistan have been working closely with health and disaster management agencies to save lives, consistent with the World Meteorological Organization (WMO) drive to strengthen early warnings and early action and to implement heat-health action plans.

We have reached, and exceeded, multiple "tipping points”, beyond which options for stabilizing the environment become more and more difficult ( Lenton *et al.*, 2019). As man-made global warming contributes to melt the polar ice sheets, less sunlight is reflected from Earth to outer space. This means that the planet absorbs more heat, temperatures rise even higher, and the ice melts even faster. Once this feedback loop crosses a critical threshold, it will gather an irreversible momentum and all the ice in the polar regions will melt even if humans stop burning coal, oil and gas. If the only small glaciers in Africa, atop Mt. Kenya and Kilimanjaro, disappear, the rivers they feed with water will run dry, affecting all life in large regions of eastern Africa.

***Climate adaptation, mitigation and resilience.*** Rising greenhouse-gas emissions could soon outstrip the ability of many communities to adapt. An IPCC report by more than 270 researchers from 67 countries finds that the negative impacts of climate change are mounting far faster than scientists predicted just a few years ago ( Tollefson, 2022a); ( IPCC, 2022a). Many effects are unavoidable and will hit the world’s most vulnerable populations hardest, it warns; this is already seen in the IPCC’s 6 ^th^ Assessment report, which states categorically that, “any further delay in global action on adaptation and mitigation will miss a brief and rapidly closing window of opportunity to secure a livable and sustainable future for all” ( IPCC, 2022b).

### The IPCC and the global context

***The IPCC.*** The climate emergency is a political issue as much as a scientific one. The urgency of the situation led to the establishment of the Intergovernmental Panel on Climate Change by UN General Assembly Resolution No. 43/53 in December 1988. Recognizing the need for governments to take an informed lead in protecting their citizens from the impacts of the climate crisis, the IPCC was tasked with preparing a comprehensive review and policy recommendations on the state of the science of climate change, its social and economic impacts, and potential response strategies. Since then, the IPCC has conducted six assessment cycles and delivered various assessment reports covering these three areas.

The first assessment report (AR1 ( IPCC, 1992)) resulted in the creation of the UN Framework Convention on Climate Change (UNFCCC ^2^), an international treaty to reduce global warming and cope with the consequences of climate change. The 191 signatory countries represent three groups according to their level of greenhouse gas production. Highly industrialized nations, OECD countries and LMICs. Each nation has committed, through the 2015 UN Paris Agreement adopted at COP21, to report every five years on their Nationally Determined Contributions (NDCs): their efforts to reduce national emissions and adopt their climate change adaptation strategies (targets, policies and measures adopted) with the joint goal of keeping the increase in global average temperature to well below 2%C above the pre-industrial level. NDCs also describe the need for research, finance, technology and capacity-building to reach their respective goals. 

The IPCC, through various working groups, monitors and publishes progress in science and national implementation. The IPCC synthesis report in 2021 indicated that there has been some progress in limiting emissions but there is still much stronger commitment and effort required. The starkest message yet of the IPCC, delivered in its 6th Assessment Report (AR6) ( IPCC, 2022a), emphasizes that the window is closing rapidly ( Tollefson, 2022a) and governments are acting too slowly, held back by the lobbying efforts of fossil-fuel companies, in turn, resulting in the continued rise of greenhouse gas emissions. Wealthy countries are responsible for causing the most global damage to the detriment of least wealthy countries. Yet, as of 2021, instead of reducing greenhouse gas emissions (other than a brief lull in 2020 due to the Covid-19 pandemic), the global emissions rate continues to increase due to the worldwide addiction to fossil fuels. Hence it is not enough that we recognize the danger: we must do something now.

***Is “green growth” possible in Africa?*** Although there’s now consensus that negative environmental impacts of human activity are irreversible, researchers disagree on the solutions, especially where they involve curbing economic growth to the detriment of making progress against this urgent challenge. One viewpoint, dubbed “green-growth,” is that economies can grow without making the planet unlivable ( International Institute for Sustainable Development (IISD), 2005). Advocates of this position point to evidence that economies continue to expand even as technological solutions, such as renewable energy, enable a gradual reduction of carbon emissions. This argument says that the solution lies in continued economic growth coupled with the acceleration of green technological solutions. A parallel approach, known as “post-growth” or “degrowth,” says that the world needs to abandon the idea that economies must keep growing because growth itself, as traditionally measured by GDP, is harmful ( Daly, 1996). However, governments worldwide have entire departments dedicated to ensuring that GDP always points upwards… and that is the problem, say post-growth researchers: when governments globally are faced with a choice between policies that promote growth or protect the environment, they are incentivized to favor growth because productivity is measured in GDP.

*A World Health Organization (WHO) report points out that if policymakers didn’t have a “pathological obsession with GDP”, they would spend more on making health care affordable for every citizen. It points out that health spending does not contribute to GDP in the same way that, for example, military spending does, making military spending more compelling than spending on health when the outcome metric is GDP ( Mazzucato *et al.*, 2022).*

The opportunities for cooperation between these camps are obvious and urgent. Both green-growth and post-growth scientists and policymakers need to see the bigger picture. There is the material risk that this continued philosophical conflict results in the delay of decisive action by policymakers. For Africa’s economic growth to be sustained, the continent must grapple with a massive infrastructure deficit, poor management of her vast natural resources, unprecedented urban growth, the challenges associated with ensuring food security and perennial extreme climate events that impact all sectors of modern day society ( Sperling *et al.*, 2012). Most of today’s energy solutions are extractive and considered from a point of view that does not proportionately account for the African context. The Science for Africa Foundation is positioned as a leading voice for the science of climate change in Africa, producing sound and relevant climate research and innovation upon which the continent’s scientists can build solutions that consider Africa’s unique context and most effectively minimize the damage caused to Africa and the world by climate change.

*Some saw it coming. Fifty years ago, the System Dynamics group at the Massachusetts Institute of Technology had a stark message for the world: continued economic and population growth would deplete Earth’s resources and lead to global economic collapse by 2070 ( Meadows *et al.*, 1972). At the time, this was a shocking forecast, and it did not go down well.*

### The impacts

***An increasingly toxic global environment.*** Humanity is becoming more aware of the myriad ways in which we disrupt the delicate ecological balance that has been shaped over millions of years. This is a merciful step towards doing something about it individually and collectively. For example, in small quantities, phosphorus is an essential nutrient for fertilizing plants, but it becomes toxic in modern industrial farming where fields are artificially fertilized by inundating them with phosphorus. Up to 80% of mined phosphorus is used in making fertilizers. The run-off poisons rivers, lakes and oceans, with devastating impact on marine and aquatic life.

Even ecosystems that produce little toxicity suffer from the damage of global toxins. In Africa, habitats are degraded and are increasingly being lost, animals and plants are becoming extinct, entire ecosystems, such as the Sahel region, are facing destruction, biodiverse marine coral barrier reefs are being bleached and the equatorial rain forests of the Congo, known, with the Amazon, as one of the “lungs of the world,” are under siege. If humanity continues its present course, the result will be not just annihilation of a large percentage of all life forms, but the destabilization of human civilization.

One third of the world’s greenhouse gases result from today’s agricultural practices ( Gilbert, 2012). Africa’s agricultural sector, for example, is heavily dependent on synthetic fertilizers. Worldwide agriculture and food production relies on synthetic fertilizers. By far, the largest producer of synthetic fertilizers is China, at 30,000 metric tons annually, followed by the USA, India and Russia whose combined annual production equals China’s. The production of fertilizers relies on a nitrogen fixation process, called the Haber-Bosch process that outputs ammonia and greenhouse gases e.g. nitrous oxide, and consumes 1% of energy globally contributing up to 1.5% of greenhouse gas emissions globally ( Capdevila-Cortada, 2019). Fertilizers will continue to be the mainstay of agriculture in Africa but there is a urgent need for greener solutions.

***Deforestation.*** Africa’s forests are threatened by deforestation that is weakening Africa’s ecosystems’ resilience and ability to adapt to climate change and negatively impacting rainfall patterns across the continent ( Duku & Hein, 2021; Taylor *et al.*, 2022). Up to 4–5 million hectares of forest cover are lost annually ( Adedire, 2002). Forests also have an important role in cooling land surfaces, generating rainfall and removing carbon dioxide and other greenhouse gases from the air. They also serve as “water towers” storing water during the rainy seasons and allowing ground water sources to be replenished gradually through the dry seasons. Unprecedented deforestation in Kenya’s Rift Valley system has caused a worrying increase in the size of lakes and areas of Lake Victoria and the Rift Valley Lakes of Turkana, Logipi, Baringo, Bogoria, Nakuru, Solai, Elmenteita, Naivasha, Ol-Bolossat, Magadi, including Turkwel Gorge Dam and the flood plains of Ewaso Nyiro South ( Government of Kenya & UNDP, 2021). Devastating floods ravaged these areas for months from 2019 – 2020 causing landslides, lake siltation, loss of biodiversity and flooding that extended into the rainy season and displacement of populations. Lake Baringo, a freshwater lake and Bogoria, a salty, alkaline lake, came dangerously close to merging and this would have had devastating consequences on the different ecological systems of the two lakes.

***Food insecurity.*** Food insecurity is a major crisis for Africa; we must pioneer new and better, climate-smart ways to combat it. At the 2021 UN Climate Change Conference (COP26) in Glasgow, world leaders pledged billions of dollars for sustainable farming and agricultural research. This commitment comes at a critical time for Africa as climate change, through unpredictable and extreme weather, is wrecking harvests. Farmers are dependent on regular seasonal rainfall, but it is becoming more unpredictable and unreliable, being either too heavy or lacking in eastern and western Africa where, for example, swarms of locust have devastated crops and livelihoods have been destroyed. Hunger is on the rise across the continent with more than one third (278 million) of the world’s people living with hunger being in Africa ( FAO *et al.*, 2022). Africa’s farmers need support to adapt to this seasonal variability. The SFA Foundation will contribute through partnerships that strengthen agriculture and biosecurity R&D systems.

***Loss of biodiversity.*** There is huge potential in Africa for developing a greater understanding of longer timescale mechanisms and events that have shaped and may continue to define the survival of mankind. Combined genomic, palaeobiological and environmental studies can inform long term strategies for conservation and improvement of food systems. The Human Heredity and Health in Africa (H3Africa) initiative facilitates the competitiveness of African researchers in human genetics and genomic sciences by supporting research on populations in Africa and in the African context, developing infrastructure, resources, training, and ethical guidelines. The initiative consists of 51 African-led research projects on population-based genomic studies of common, non-communicable and communicable disorders. This work has contributed significantly to understanding the underlying genetic and environmental contributions to risks and vulnerabilities of these diseases across Africa’s genetically diverse populations.

### Climate change implications for Africa

***Interdisciplinary research innovation and training.*** Africa is disproportionately burdened by multiple and cascading impacts of climate change. The challenges are complex given the needs for economic development (driven by fossil fuels in most countries) and the expansion of infrastructure needed to support it, unprecedented population growth and urbanization, increasing pressure on the continent’s environment and ecosystems. Investments made for global adaptation do not prioritize Africa’s needs and there is a significant knowledge gap in the understanding of the science of climate change and sustainable solutions for adaptation. The spatial and temporal gaps in observations of climate and weather are a serious impediment to the research on climate modelling and research that is specific to understanding Africa’s complex ecosystem is limited. Without a healthy science environment, Africa will continue to struggle in efforts to adapt to climate change.

***Extreme events.*** Reports from the IPCC and the UK Meteorological Office indicate alarmingly that by 2019, about two billion people had suffered from extreme heat and 356,000 died from heat ( Tuholske *et al.*, 2021). In 2021, life-threatening heat waves were experienced in the Sahel, reaching over 40°C, and similar levels have been recorded in India in 2022, with temperatures in Delhi reaching 49.2°C ^3^. Many regions, particularly big cities, continue to get hotter. In Africa’s largest cities, Lagos (Nigeria), Maputo (Mozambique) and Omdurman (Sudan), hot days can kill, and even uncomfortably hot nights can affect health, with the poorest people most affected.

In recent years, such extreme weather and climate events are increasingly palpable in Africa: increasing desertification; disappearing ice caps; rising oceans and more frequent; extreme weather events such as heatwaves, hurricanes, typhoons in Mozambique and Madagascar and wildfires in Algeria. Wildfires in Africa amount to 67% of the area of global fires ( United Nations Environment Programme (UNEP), 2022b). In addition, these changes are already disrupting agricultural production – the eastern Africa region has suffered four consecutive years of failed rainy seasons, causing severe drought for humans and animals. 3.5 million head of livestock and wild animals have perished during this period in the pastoral areas of Kenya, Ethiopia and Somalia due to lack of water and pastures.

Weather extremes also manifest in flooding. In early 2022, rains inundated the Kwa-Zulu Natal region of South Africa. More than 500 people perished, and 40,000 were left homeless by mudslides. Parallel events played out in Europe, South America and elsewhere in 2021/2022. Climate change is increasing the proportion of the world that is uninhabitable and sending hundreds of millions of refugees in search of new homes, adding to the ongoing poverty-driven migration out of Africa, regional conflict and a mounting refugee crisis for governments hosting displaced communities.

***Pollution.*** Pollution kills nine million people a year and one in six deaths worldwide in 2019 were related to pollution ( Fuller *et al.*, 2022). Almost all of these (90%) occurred in low and middle-income countries. Fortunately, deaths attributable to the types of pollution associated with extreme poverty, such as poor sanitation and household air pollution caused by burning fuel indoors, are declining. But newer forms, including the effects of particles from burning fossil fuels and lead from poor recycling practices for batteries and electronic waste, are on the rise. On balance, the situation has not improved since 2015.

How pollution, poor ventilation and lack of clean energy affect health and wellbeing in Africa is known: higher air pollution levels increase short-term respiratory infections leading to chronic obstructive pulmonary disease (COPD), compromising not only physical health but also education and livelihoods through increased school and work absences. Household air pollution is associated with major health effects, especially in women and young children, who stay indoors for longer periods. Workers on traffic-congested roads and children who play outdoors and live in highly polluted areas are more likely to develop asthma, as are those who live near busy roads. Asthma sufferers who have been exposed to high levels of air pollutants are more likely to develop bronchitis and exacerbation of existing disease such as TB. Higher pollution levels can cause lung damage. Air pollution exposure is associated with oxidative stress and inflammation of human cells, which may lay a foundation for chronic diseases and cancer. This is why the WHO International Agency for Research on Cancer has classified air pollution as a human carcinogen.

Despite the unanimous resolutions of COP26, replacing fossil fuels with clean renewable energy sources is problematic for many African countries. The economies of Nigeria, Angola and the DRC, for example, are heavily dependent on exporting oil and LPG. In fact, as reported in the New York Times ^4^, “ *The Democratic Republic of Congo, home to one of the largest old-growth rainforests on Earth, is auctioning off vast amounts of land in a push to become ‘the new destination for oil investments’*.” It furthermore attributes to Tosi Mpanu Mpanu, the country’s lead specialist on climate issues, the blunt and chilling assessment that, “ *our priority is not to save the planet.”*

Even African countries that import oil and gas don’t have sufficient resources to build their own renewable solar, wind and geothermal infrastructure. Thus there is no consensus among African governments on goals for reducing global carbon emissions as soon as possible (by 2030/40). Even countries who signed COP26 agreements to limit emissions to achieve no more than a 1.5°C rise in temperature, both wealthy countries and LMICs, will be subject to shorter-term economic and political pressures to delay or abandon their commitments. ( *e.g*., Botswana has commissioned a new 1.4 million ton-per-year coal mine driven by global demands in the wake of the Russia-Ukraine war ^5^). 

***Conflict.*** Climate change is driving existing and emerging conflicts in Africa. Peacekeeping and climate change should be a priority for Africa by recognizing how conflicts can be driven by the impact of climate change on the continent: when icecaps diminish, rivers dry up and generate conflicts over lack of water. Water is vital of course for human and plant survival and to enable grazing to support livestock that are the only source of income for many people. Fighting over access to resources that enable people to earn income has emerged most acutely in the pastoral communities of the Horn of Africa.

This crisis promises to persist. If the only three glaciers in Africa cease to stock rivers with water, what does this mean for the eastern and central African regions? Will it bring a drier savannah/desertification for humanity and all biodiversity? If the rivers stop flowing, the inevitable result will be conflicts between and within communities, particularly pastoralists who are dependent on water towers. In other places, drought has been exacerbated by climate change and terrorism (e.g., Somalia, the Sahel belt), and people are fleeing to seek refuge across the globe. In the Lake Chad basin, where stress on available water has promoted conflict, disease outbreaks have been reported for many decades in Sudan, Central African Republic, DR Congo and other areas. Disease incidence is very likely worse than is reported. COVID data from many countries is understated mainly because of the inaccessibility of many communities and little capacity to collect and report data. The DR Congo has reported fewer than 1500 cases annually of active Monkeypox for many years, but there is no doubt this is a huge underestimate.

*“We (humans) are one species, we came out of Lake Turkana three million years ago and we have to recognise we're all in the same house. I am full of confidence that we have the human resources; Africa has just got to be given a little bit of a lift... to stop the habitat loss and human expansion to encroach on water systems and forests and pollute the air." **Richard Leakey, Kenyan fossil expert, author and conservationist.***

### Priority areas for a green transition in Africa

***Alignment with global priorities and strategies.*** All fifty-four African countries signed the 2015 Paris Agreement and an analysis by the European Centre for Development Policy Management (EDCPM) indicates that green transition is gaining traction in Africa ( Ashraf & van Seters, 2022). There is an urgent need to boost economic growth across all sectors in Africa and the pathway towards a green economy needs to be supported and paced to allow the continent to build her economies, capacities and policy environments while considering climate change and environmental issues. The choices governments make in the key sectors of energy, biodiversity, agriculture and health will be dependent on locally relevant scientific evidence.

Africa’s scientists need to play a key role in collating locally relevant evidence, performing assessments, leading the African discourse in global reporting and analysis processes and providing evidence and information for governments that enables policy level decision making. It is time for the continent’s scientists to engage with policy makers to collaboratively drive a sustainable socio-economic growth pathway by seeding green start-ups, creating high value green jobs and nurturing a sustainable circular economy at the core of the green transition in Africa.

The SFA Foundation’s strategy for climate change is informed by the priorities, principles and recommendations of AU/AUDA-NEPAD, the IPCC, the EU-Africa Advisory Board, FCDO’s Adaption Research Alliance (ARA), the Green Transition & Leadership for Africa Program (GTLAP), the WMO world climate research programmes and other thought-leader partnerships. These strong continental and global collaborations can stimulate a green innovation transition for Africa that will address climate change and environmental degradation for our local context in the key areas of energy, biodiversity and agriculture.

***Renewable energy.*** Some governments have taken up the challenge and there are success stories in the energy sector across the continent. The Nigerian government is taking steps to expand renewable energy generation, including solar energy. Kenya is one of the few countries to develop geothermal energy. Seventy-three percent of Kenya’s energy supply is from renewable sources and 27% of this is from geothermal supply. Morocco has a massive potential for renewable energy, including natural gas, wind and solar. Renewable energy supply is on target to reach 52% by 2030 there. South Africa’s industrial sector is heavily driven by fossil fuels and contributes to half of Africa’s greenhouse gas emissions. The government of South Africa has signed agreements with partner nations to close down coal mines and transition to clean energy sources.

***Evolutionary biology and biodiversity loss.*** Africa’s unprecedented growth in population, urbanization and agricultural development presents a significant challenge in balancing economic prosperity and human wellbeing with ecosystem and environmental preservation. The loss of Africa’s rich species diversity is unprecedented. Signatory countries to the UN Convention of Biological Diversity have agreed to meet global targets for biodiversity ( UNEP-WCMC, 2016). Notable among these is to halve the rate of loss of all natural habitats, to prevent the extinction of threatened species, and to eliminate harmful subsidies and economic incentives that have a negative impact on biodiversity and ecosystems. The pressures on Africa’s economies make the targets difficult to meet. Africa continues to lag behind global progress in improving knowledge on biodiversity (Target 19) and making necessary financial resources available (Target 20).

For decades, Africa has been a world leader in ground breaking studies of evolutionary biology. Over the last 50 years, discoveries of many hominin fossils, from Eastern and Southern Africa, have been featured as cover headlines on more than 10 Nature publications ( Asfaw *et al.*, 2002; Barash *et al.*, 2022; Brown *et al.*, 1985; Martin *et al.*, 2020; Roche *et al.*, 1999; Spoor *et al.*, 2007; Walker *et al.*, 1986). This rich fossil heritage is poorly celebrated in Africa, including in paleoclimatology - the study of how climate impacted early hominin life in the geological past. Palaeoclimatology has greatly increased the understanding of climate change in the past and aided the prediction of its possible impacts in the future ( Crowley, 1989) including the Green House effect and how ice ages start or end, the impact of ozone levels in the past and its impact on local ecologies and species.

Understanding our past puts us in a better position to predict our future. Locally (Kenya, South Africa, Egypt, Sahel regions), and globally, African scientists are working to uncover clues to the causes of natural droughts to forestall the damage of future climate change to food security and health. These approaches and methodologies can be vital for understanding how our world is changing over oceans, air and land.

The SFA Foundation will support the career development of emerging scientists in paleoclimatology and species biodiversity who also address climate and health challenges in specific areas of anthropology, ecology, evolution and geosciences, as well as more granular areas such as morphology, taxonomy, stratigraphy (the study of fossils and their use in dating rock formations) and studies of tree ring data in extinct forest marginal zones, ice cores, sediments, zoology remnants (such as horns, shells and bones) and coral reefs. These can all reveal indicators of past climate variables such as air quality and makeup that are impacting current climate and health challenges. These scientists require access to good labs, infrastructure and offices, locally and internationally; they also must collect samples to analyse in the field. The SFA Foundation can help build such careers and infrastructure in Africa. For example, the Kenya-based Turkana Basin Institute, founded by the late world-renowned Richard Leakey, could be a model for similar palaeontology centres in Africa, potentially in partnership with international academic institutions. 

***Coalitions in genetics and genomics.*** There is an urgent need, driven by the rapid loss of biodiversity in Africa, to collate a record of this biodiversity by genomic sequencing of eukaryotic species. The African BioGenome Project (AfricaBP) has assembled African scientists in an effort to achieve this ( Ebenezer *et al.*, 2022). The SFA Foundation has partnered with the researchers of the AfricaBP in their effort to sequence the genomes of 105,000 endemic plants, animals, fungi, protists and other eukaryotes. AfricaBP calls on agencies and organizations to allocate long-term investments to the project. “This store of reference genomes — built in Africa, for Africa — will help plant and animal breeders to produce resilient and sustainable food systems…It will inform biodiversity conservation across the continent. And it will strengthen Africa’s ability to deliver on the goals of the post-2020 global biodiversity framework of the Convention on Biodiversity ( Ebenezer *et al.*, 2022).” In its mission to further African research capacity in environmental genomics, AfricaBP has engaged 109 African scientists (87 of whom are based in Africa) and 22 African organizations. Given Africa’s vulnerability to these threats, these areas of science need to be led by African scientists to ensure that they address local priorities for sustainability.

**African BioGenome Project Research Priorities**

- Study optimal preservation of the biodiversity of plants, animals, fungi, protists, and microbes present in our ecosystems, which also directly deliver on global SDGs.
- Coordinate existing non-human genome sequencing initiatives across Africa as an SFA Foundation/AfricaBP partnership.
- Support new non-human genome sequencing across Africa.
- Deploy sequencing data to regional biobanks and other continental agencies, including the African Union Inter-Africa Bureau for Animal Resources (AU-IBAR), to inform policies on biodiversity and build capacity in genomic sciences.
- Research on how to leverage biodiversity in Africa to deliver interventions that address climate challenges, in turn strengthening Agri-food systems and sustainable health interventions.
- Advocate for the protection of an African biodiversity landscape as a shared global heritage for current and future generations.

***Afforestation.*** Tropical forests play a crucial double role in cooling the Earth’s surface: two-thirds of the forests’ cooling power comes from their ability to suck in and store CO _2_; the other third comes from their ability to create clouds, humidify the air and release cooling chemicals ( Kreier, 2022). The biophysical effects of the world’s tropical forests are to collectively cool the surface of the planet by around 1°C ( Lawrence *et al.*, 2022).

More than three-quarters of the Amazon rainforest is losing its ability to recover from fire and drought ( Staal *et al.*, 2020); although there are less data on the Congo/African equatorial forests, the pattern is similar. This degeneration could push the world’s largest rainforests past a tipping point where the forests could convert to an open savannah mix of wood and grassland with horrific consequences for biodiversity and the global climate ( Boulton *et al.*, 2022).

*The generation that destroys the environment is not the generation that pays the price…that is the problem…. You can make a lot of speeches, but the real thing is when you dig a hole, plant a tree, give it water, and make it survive…that is what makes the difference* ( ***Wangari Maathai, Nobel Laureate***).

Over centuries, the indigenous peoples of the Congo/Equatorial basin and other forest areas have done the hard work of on-the-ground conservation, and now more than ever, must be empowered. Oil spills in the Nigerian Delta regions remain a global example of how big, profit-driven extraction companies damage the environment and marginalize indigenous people. There is also illegal logging when it is the only source of subsistence and domestic usage. Governments must provide alternative sustainable resources and livelihoods to indigenous peoples who resort to this activity to survive. Where central governments fail to reach indigenous forest guardians, the slack must be taken up by vigorous biodiversity preservation through monitoring of wildlife populations, fisheries and oil spills.

About 30% of residents of Africa’s big cities have no access to electricity and cannot buy simple cooling fans for their homes. The easiest way to tame heatwaves, and consequent heat stroke and dehydration, is to plant more trees and limit fossil fuels. Greening cities, changing labour laws to protect those who work without cooling, and redesigning buildings can all help. Beyond these global actions, individual actions, such as green choices for household energy, transport, lifestyles, diet, food production, personal transport and management of waste, promote a circular economy. For example, scientists calculate that replacing just one-fifth of global beef consumption with a meat substitute within the next 30 years could halve deforestation and the carbon emissions associated with it. Researchers modelled the effects of swapping beef with a fungus-based meat substitute called mycoprotein, familiar to many as Quorn. Replacing 80% of beef other food sources would eliminate about 90% of forest loss.

The fight against destruction of Africa’s forest areas is challenged by competing national priorities, poor governance and lack of commitment. The *New York Times* reports that leaders of central African countries backed out of a deal with conservation groups to compensate them for protecting their forests, which are also the world’s most important sanctuaries for gorillas, as the Russian invasion of Ukraine sent global oil prices higher. Congo’s lead representative on climate issues, Tosi Mpanu Mpanu, declared, “our priority is not to save the planet”. His blunt words highlight a crucial question for the climate emergency: how can rich countries, which built their prosperity on fossil fuels and the exploitation of other regions’ natural resources, demand that poorer nations keep their fossil fuels in the ground? “Maybe it’s time we get a level playing field and be compensated,” said Mpanu. “We just have to see how much people value that resource.”

Fifteen years ago, the African Union committed to Africa’s Great Green Wall, a staggeringly ambitious initiative, spanning 8,000 kilometres, from Senegal to Djibouti, and one of the world’s biggest ecological-restoration schemes; aiming to restore 100 million hectares of degraded land by 2030. It was projected to enable restored forests to capture 250 million tons of carbon dioxide; and create ten million jobs.

In two assessments, independent experts commissioned by the United Nations and the UN Convention to Combat Desertification ( UNCCD, 2022), indicate that only 4–20% of the forest restoration target has been met. The shortcoming, not surprisingly, is attributable to perennial shortages of funding; so far, $19 billion has been committed, but the funds are not flowing into the project regularly. Africa’s governments and international donors are about US$30 billion short of the 100-million-hectare target. There are also political and accountability challenges. Although the authority for the project is assigned to the AU-established Pan African Agency of the Great Green Wall (based in Nouakchott, Mauritania), some donors choose to provide funding directly to individual governments, giving the donors more fiscal control. The project continues to struggle to achieve its goals. A Nature editorial summarizes the challenges facing Africa in recapturing historical levels of forest cover including poor commitment and a need for research ( Editorials, 2020). This research needs to be led by researchers in Africa, supported with best-practice programmatic oversight, improved outcome metrics and transparent accounting and governance to address accountability gaps. These problems can and must be fixed for Africa’s Great Green Wall and similar projects to succeed.

***Climate, environment, food systems and health.*** All climate change impacts experienced in Africa have specific and serious consequences for human health and health systems. Extreme weather events; tropical storms, floods, droughts and heatwaves, are intensifying across the continent and exerting a significant burden on unprepared health systems and compromised food systems. Millions of lives will be lost as a result of infectious diseases and emerging pandemics as they spread faster and further, and from non-communicable diseases (NCDs) such as heart disease, which is on the increase.

Spill over events, in which a pathogen, that originates in animals, jumps to humans, have probably triggered every viral pandemic that has occurred since the start of the twentieth century, including HIV, the 1918 influenza pandemic and Covid-19 ( Vora *et al.*, 2022). Three proposed landmark international health and biodiversity agreements urge decisionmakers to prioritize spill over prevention and to protect forests, especially in hotspots for emerging infectious diseases; regulate strictly the trade of live wild animals while respecting the livelihoods of indigenous peoples and local communities; improve farm biosecurity and invest in human health and economic security to reduce high-risk activities and vulnerabilities.

Incontrovertible evidence demonstrates that climate change will boost viral outbreaks. It is estimated that over the next 50 years, climate change could drive more than 15,000 new cases of mammalian transmission of viruses to other mammals when animal species cluster in cooler and more water-available locales as temperatures rise. This is especially threatening in species-rich ecosystems in densely populated, high elevation areas of Africa and Asia. As seen with Ebola, Covid-19 and Monkeypox, the stage is being set for future epidemics and pandemics that are likely to emerge from Africa or elsewhere as a result of increased proximity of infectious pathogens with humans as they cross natural ecological barriers between human-plant-animal ecosystems.

***Plastic pollution and the circular economy.*** Eliminating pollution due to plastic waste is another priority. Plastics originate from fossil fuels and the extraction and production processes generate and release large amounts of greenhouse gases into the environment. Further, if incorrectly disposed of, plastic waste emits greenhouse gases when exposed to solar radiation, negatively impacting ecosystems and climate.

The fifth session of the UNEP United Nations Environment Assembly ( UNEP, 2021–2022) noted that microplastic waste has become a major area of concern globally. Marine plastic pollution is abundant and ubiquitous and is threatening the biodiversity of the world’s oceans. The impact on coastal communities, particularly Small Island Developing States (SIDS), with economies dependent on tourism and fishing, is particularly severe. Microplastics have found their way into living organisms and are a serious threat to the health of humans and other species.

Leaders of 175 nations at the United Nations Environment Assembly (UNEA) agreed to a treaty to tackle plastic waste, details of which will be negotiated over the next two years ( UNEP, 2022a). Rwanda was one of the first countries to ban the use of single-use polythene plastic bags, and a transformative law passed in 2019 began a phasing out of all single-use plastics. Delegates rose in ovation as a resolution was adopted by Kenya to follow suit in banning plastic bag use in 2022. Crucially, the treaty will be legally binding and address the full life cycle of plastics. Inger Andersen, the executive director of the UN Environment Programme, called the agreement, “the most significant environmental multilateral deal since the (2015) Paris climate accord” ( Graham, 2022).

***SFA Foundation’s positioning and strategic partnerships.*** The SFA Foundation is a pan-African non-profit organization that funds scientific discovery, innovation and translational sciences across three key interrelated areas of science: Health R&D, Climate Sciences and Agriculture & Food Security. Its long-term strategy positions Africa as a major global player in climate change breakthroughs across fields, through a better understanding of Africa’s climate science, innovations for adaptation and creating strategies for resilience to climate threats, locally and globally. Africa’s solutions and interventions should come from Africa and this is a priority for the SFA Foundation.

The Foundation’s strategy supports *People* – both emerging talent and established leaders – by providing resources and capabilities to deliver world class scientific projects and programs; establishes independent research groups, training and mentorship for early career researchers; creates *Places* of work conducive to producing quality science outcomes and effective use of resources; encourages the development of *Products* to support innovation; translates scientific findings into *Policies* that are embraced by policymakers, governments and communities, all the while, recognizing that these impacts will only be achieved through strong and equitable *Partnerships* with African stakeholders, global partners and funders. Climate change adaptation research by the SFA Foundation prioritizes practical and implementable solutions that make a positive impact on the lives of people vulnerable to current or future climate change, and its impact on health, the environment and food systems.

A role of the SFA Foundation is to support the many partners and advocates who are calling for the building of global health efforts that are synergized by and/or complementary to climate treaties. For example, the SFA Foundation has joined the Adaptation Research Alliance (ARA), a coalition of stakeholders across the adaptation research and action communities, including traditional ‘research funders’ and ‘action funders.’ The remit of the ARA aligns with the mission of SFA Foundation - to scale up research and capacity building internationally to build locally-relevant adaptation strategies to address climate change. (Ref COP26 Adaptation & Resilience: ARA Concept Note v12 [R. West/A. Patwardhan 1 June 2020] OFFICIAL Proposed mechanisms).

The SFA Foundation will prioritize research on the impact of climate change on human health based on evidence of its potential to address urgent under-examined areas. For example, as with Covid-19 and previous epidemics that emerged from Africa (Ebola, Lassa, River Valley Fever), the SFA Foundation will also support the strengthening of health systems, surveillance, the training of infectious disease and public health experts, and product development through quality discovery research and translational and data sciences.

In 2022, the SFA Foundation, and its global funding partners, announced the Developing Excellence in Leadership, Training and Science (DELTAS Africa) program to support large African networks that are designed specifically to strengthen climate sciences and related health challenges. These networks will be built on the ONE Health framework that accounts for the interrelationships among climate-health challenges in Africa that affect soil, food systems, the movements and living conditions of people and other areas of physical and social science that examine how climate change impacts our health.

SFA Foundation will seek to join the Agriculture Innovation Mission for Climate (AIM4C) ^6^, which, together with the ARA, provides support for climate adaptive farming, agricultural research and food systems. Led by the USA and UAE governments, the AIM4C has received $4 billion in pledges, including for research aligned to SFA priorities. Other potential SFA partners include organizations that pledged significant research support at COP26, such as $1 billion by the Consultative Group on International Agricultural Research (the largest research organization assisting small scale farmers in the developing world), and the UK government who pledged $197 million by programs in Africa.

In strong partnership with AU/AUDA-NEPAD, the SFA Foundation is ideally positioned to call upon African governments to invest more money in R&D for revolutionary, eco-friendly innovations and technologies.

## Leadership and the Way Forward

The 2021 UN global Conference of the Parties to the United Nations Framework Convention on Climate Change (COP26) meeting in Glasgow was deemed a success, raising expectations that the world will reduce carbon emissions to zero. But as of February 2022, major companies are already falling short of their promises. An analysis of publicly available corporate documents shows that a cross-sector sample of 25 companies which together are responsible for about 5% of global emissions are actually committed to doing far less than promised ( Tollefson, 2022b). Few have clear blueprints for decarbonization. Thirteen of the 25 countries provide plans that would, on average, curb emissions by just 40% over the next few decades; the others have not released their plans.

To maintain a stable physical and economic environment, governments must focus on transitioning from current global-standard inventions such as internal combustion engines to manufacturing electric batteries and cars ( Tabuchi & Plumer, 2021). This is an opportunity for the DRC Congo, with its vast mineral resources including cobalt and lithium, to transition from exporting its mineral wealth to developing a domestic battery industry to be used in electric cars and cellular phones worldwide. It is imperative to accelerate the transition from usages that burn fossil fuels to sustainable technologies across economic activities for developing countries. This is a particularly difficult mandate for African countries whose economies are dependent on commodities such as oil, coal and gas. The temptation for leaders to literally sell off their natural birth right is understandable given the challenges these countries face and the pressure is increasing given the global energy scramble precipitated by Russia’s invasion of Ukraine. The green energy transition requires increased support for development of R&D based solutions.

A variety of green policies can be adopted in Africa, many of which make good economic and environmental sense. Governments must tax carbon emissions, add the cost of externalities to the price of oil and gas (Nigeria, Angola and South Africa are key producers of oil and coal), adopt stronger environmental regulations, discontinue provision of subsidies (and implement penalties and taxes) to polluting industries and transition to renewable energy. Countries such Morocco and Kenya, where over 80% of energy is generated from solar, geothermal, hydro and wind power sources, have begun to take steps in this direction.

The 27 ^th^ COP has been met with mixed reviews though there is some positive news for climate justice and adaptation. A USD100 Billion “Loss and Damage Fund” was announced and, UN Secretary-General António Guterres, described this as an “important step towards justice.” The fund responds to repeated pressure by developing nations and numerous climate activists, over 30 years of climate discussions, for commitment by the most industrialized nations to take action on their contribution to the climate crisis. However, COP27 closed with some serious lack of clarity, from the globe’s greatest greenhouse gas emitters, on targets for mitigation. Key commitments, made at COP26 in Glasgow, e.g. the target to peak emissions by 2025, made with 1.5°C above pre-industrial levels in mind, were dropped from final agreements, due to pressure from petroleum producing countries with the support of a few other industrialized nations. The wording of agreements has been considered, by some, to weaken commitment; natural gas was defined a “low emissions energy source” and the COP26 push to phase out coal was changed to phasing down coal. The influence of the fossil fuel industry is heavy and increased by the fact that most developing countries are dependent on oil, coal and gas, having limited investment in clean energy.

The COP27 presidency also launched the Sharm-El-Sheikh Adaptation Agenda, a global plan to rally all around 30 adaptation action outcomes. The adaptation agenda is a call to global efforts to amplify climate financing, technology transfer and capacity-building for adaptation in response to the needs of developing country Parties. Its outcomes on climate resilient agriculture, biodiversity conservation, access to clean energy, innovative climate financing and climate risk planning by large companies, are of particularly importance to climate adaptation in Africa. The COP27 Presidency announced its support to all adaptation focused initiatives launched by COP26, including the Adaptation Research Alliance (ARA) that celebrated the successes of its first year. The ARA is a UK government and IDRC supported global collaborative effort 2030 in which the research community is a highly valued partner to policymakers, practitioners and the most vulnerable communities, and engages effectively to support the delivery of innovative, user-driven solutions for adaptation and resilience from the global to local levels. ARA’s mission in the shorter term is to accelerate and scale investments in action-orientated research in developing countries. ^7, 8^

Other key discussions on biodiversity have stalled because of disagreements over how much funding must flow from developed nations ( Gilbert, 2022), whose consumption drives biodiversity loss, to low- and middle-income countries, many of which are home to areas rich in biodiversity but don’t have the means to conserve them. There is hope that the United Nations biodiversity summit (2022) will produce progress. ^9^

To fund the necessary initiatives, the world’s 20 largest national economies have committed to include climate action, including “green new deals” and “building back better”, in their pandemic economic recovery packages, which total US$14 trillion. So far, these commitments have not been met ( Nahm *et al.*, 2022): only 6% of total stimulus spending (about $860 billion) has been allocated to areas that will reduce greenhouse gas emissions - proportionately less than the green investments that followed previous recessions.

There are hopeful signs from the United States, China, South Korea and regions such as the European Union that money can be invested, and that can serve as a model for other countries. However, the human suffering and death caused by Russia’s invasion of Ukraine will complicate global cooperation on climate change and the transition to greener energy, by forcing some European countries to become more reliant on coal in the short-to-medium term, as they work to break their dependence on Russian gas and oil. Despite there being significant buy-in and progress among African countries in meeting their Paris Agreement commitments, there are still significant gaps and challenges related to weaker institutional frameworks (appropriate delegation of authority with sufficient coordination and collaboration platforms for inter-ministerial engagement), availability of robust policies, systems and processes for monitoring, reporting and verification of emissions and monitoring and evaluation (M&E) of adaptation, climate finance and technical support outcomes, as well as open data and information sharing platforms which promote accountability and transparency. This could be a possible area of interest for intervention of SFA as part of the institutional strengthening area of work.

There is good news from the IPCC report; i) renewable-energy technologies needed to make the change are available and are increasingly more affordable; ii) It is possible to pull carbon pollution out of the atmosphere by expanding forests and improving agricultural practices, and iii) the economic benefits of limiting warming, including improved health and reduced damage, exceed the cost of mitigation. We now must seriously align our political and economic priorities to dedicate resources to impactful scientific research, from basic cellular research to the study of human and animal behaviour and strengthen our own African science base while working with other countries to tackle the existential crisis of climate change. African countries will have to punch above their weight in order for these global efforts to prevail. With trusted partners in science, we can hope for a more secure and sustainable future as we target our science towards delivering impactful solutions.

## Data Availability

All data underlying the results are available as part of the article and no additional source data are required.

## Footnotes

^1^ https://www.worldweatherattribution.org/

^2^ UNFCCC: United Nations Framework Convention on Climate Change

^3^ https://www.bbc.com/news/world-asia-india-61242341?utm_source=Nature+Briefing&utm_campaign=41b064178b-briefing-dy-20220428&utm_medium=email&utm_term=0_c9dfd39373-41b064178b-46814086

^4^ https://www.nytimes.com/2022/07/24/world/africa/congo-oil-gas-auction.html. (Lead Climate Change Specialist of the Ministry of Environment, Conservation and Tourism of the Democratic Republic of Congo and Climate Vulnerable Forum Thematic Ambassador on Renewable Energy)

^5^ https://www.reuters.com/markets/commodities/botswanas-morupule-aims-boost-coal-output-by-50-with-new-mine-2022-09-28/

^6^ https://www.aimforclimate.org/

^7^ https://ukcop26.org/adaptation-research-alliance-ara-joint-statement-on-launch-9-november-2021/

^8^ https://ukcop26.org/adaptation-research-alliance-ara-joint-statement-on-launch-9-november-2021/. Adaptation Research Alliance (ARA) joint statement on launch-9 November 2021

^9^ https://www.unep.org/events/conference/un-biodiversity-conference-cop-15
