# Supplementary material for: The interconnected global emergencies of climate change, food security and health: a call to action by the Science for Africa Foundation
Source: Open Res Afr. 2023 Jan 3;6:1. [Version 1] doi: 10.12688/openresafrica.13566.1 (PMC9958300; doi:10.12688/openresafrica.13566.1)
Supplement: Peer review history

# Peer review: Reviewer response for version 1

**DOI:** 10.21956/openresafrica.14719.r29493

**Published:** 2023-02-20

**Authors:** Bassirou Bonfoh

**Affiliations:** 1 Afrique One, Centre Suisse de Recherches Scientifiques en Côte d’Ivoire, Abidjan, Cote d'Ivoire

The paper from Kariuki *et al.*, addresses the great challenge of climate change that most African people face. The paper positions Africa as the continent where the consequences are mostly observed and its population impacted in the area of food and health. The authors argue that in Africa it is the failure to prioritise climate change and not given too much attention and led to knowledge gap on the phenomenon and how to better adapt. Furthermore, it appears that part of the solution should come from where the consequences are the most seen. By considering the drivers, the rest of solution or mitigation options should also come from where others create conditions leading to climate change. The authors proposed to avoid depending solely on external funding. The authors proposed to shift the science of climate to Africa with local responses to where the impacts are important. The call of SFA for action is commended and need more research capacity with an African perspective and partnerships with all other institutions working of the same objectives. The position of SFA on the climate issue is well documented. Authors should thus show the exact niche that SFA is taking to transform the climate change knowledge.

 The paper is well written and contains the main aspects related to the topic. I suggest valuing or considering the local investments already struggling with adaptations alongside global investment in the carbon reduction. Finally the causality between climate change and some of the human conditions as food security and health is evident but needs more research to show the non-linearity links.

1. The paper lacks a chapter or short methodology on how the review has been made. This could clarify how the information and different bodies of knowledge have been harnessed and help to better structure the paper.
2. The paper is not well balanced and lacks enough information on the health and food systems as far the emerging diseases and the transformation of food system are concerned. More have been said on biology and biodiversity, energy, pollution, conflicts. Only a small chapter described climate, environment, food system and health.
3. The paper structure contains all information needed but it is hard for the reader to follow. I suggest if possible to have the following move (i) the main drivers with the African perspective, (ii) the consequences and impacts with an emphasis on food security and health as the main African challenges to address; (iii) options for responses derived from all the COPs and different gaps in term of funding, research, capacity to address the climate problem in Africa; (iv) how the SFA and other partners, with their leadership are planning to curve the impacts especially with the science of climate that is promoted. I also suggest looking at the potential of certain neglected disciplines in Africa when addressing the climate change issues such as social sciences, economics to better bring the communities voices for policy makers to understand.

Does the article adequately reference differing views and opinions?

Yes

Are all factual statements correct, and are statements and arguments made adequately supported by citations?

Yes

Is the Open Letter written in accessible language?

Yes

Where applicable, are recommendations and next steps explained clearly for others to follow?

Yes

Is the rationale for the Open Letter provided in sufficient detail?

Yes

Reviewer Expertise:

Veterinary epidemiologist, Food system and risk analysis, complex health threats at human-animal-environment interface

I confirm that I have read this submission and believe that I have an appropriate level of expertise to confirm that it is of an acceptable scientific standard.

# Peer review: Reviewer response for version 1

**DOI:** 10.21956/openresafrica.14719.r29492

**Published:** 2023-02-01

**Authors:** Cush Ngonzo Luwesi

**Affiliations:** 1 Economics and Environment, Integrated Water Resources Management, University of Kinshasa, Kinshasa, Democratic Republic of the Congo

**Major corrections:**

 This is a very good paper but many paragraphs have no or missing references; they need to be added (e.g. pages 5, 6, 7, 8, 9, 10, 12). New York Times was quoted twice but without references. 

 These concepts should be properly referenced: green growth and circular economy (P. 5, 9), loss of biodiversity (P. 6, 8), conflict on resources (P. 7), pollution (P. 7), renewable energies (P. 8), indigenous conservation (P. 9), viral outbreaks links with climate change (P. 10), CoP26 and CoP27 (P. 11, 12) should be properly referenced using a single style like APA (Author, Year) or MLA (marginal references under the page). Stick to one style; do not mix both of them, please.

 Regarding “Conflict on resources”, kindly refer to the Mbororo pastoralists migration from Lake Chad Region to the Congo Basin, thus exacerbating local agricultural activities.

 With regard to “Pollution”, kindly refer to the recently published SEI’s “Africa Assessment Report on pollution and climate change”.

 I also suggest that SFA contributions to the climate change discourse be aligned to the “SFA Foundation position and strategic partnerships” (Page 11). So, the authors may swap all of these contributions from different paragraphs and bring them under this section. This will show the difference between the background information on climate change and contributions of the SFA Foundation to this discourse.

**Minor corrections:**

- The Meadows report (1972) referred to on page 4 points to 2070: This is a “prediction, not a forecast”.
- Also replace 2%C on page 5 with 2 ^o^C.
- On page 13, remove the full-stop on “warming.”.

Does the article adequately reference differing views and opinions?

Partly

Are all factual statements correct, and are statements and arguments made adequately supported by citations?

Yes

Is the Open Letter written in accessible language?

Yes

Where applicable, are recommendations and next steps explained clearly for others to follow?

Partly

Is the rationale for the Open Letter provided in sufficient detail?

Yes

Reviewer Expertise:

PhD Appl. Sc. (Economics of water and climate change)

I confirm that I have read this submission and believe that I have an appropriate level of expertise to confirm that it is of an acceptable scientific standard.

# Peer review: Reviewer response for version 1

**DOI:** 10.21956/openresafrica.14719.r29489

**Published:** 2023-01-23

**Authors:** Tufa Dinku

**Affiliations:** 1 International Research Institute for Climate and Society, Columbia Climate School, Palisades, NY, USA

This is a well-written and well-structured manuscript that describes the challenges climate change poses to Africa, the challenges in adapting to climate change, and what needs to be done. The first two are covered very well in the manuscript. The last part, what needs to be done going forward, is not very clear to me. The last section of the manuscript, *leadership and way forward*, would need to focus on specific actions that can be taken by the Africans (including SFA) and the international community. The reader should be able to go away with a concrete message/action point. As it stands now, this section reads as a continuation of the previous sections. Many of the points discussed in this section could be moved to the previous sections.

 The manuscript rightly mentions that “spatial and temporal gaps in observations of climate and weather are a serious impediment to the research on climate modelling and research”. It would also be good to add that there are challenges in accessing existing climate data from National Meteorological Services in Africa, and promote (maybe as an action item in the last section) the idea that climate data should be a public good.

 There are many statements/claims, including numbers, without any references.

 Finally, the manuscript could be shortened as some of the background material could be assumed to be known to the reader.

Does the article adequately reference differing views and opinions?

Yes

Are all factual statements correct, and are statements and arguments made adequately supported by citations?

Partly

Is the Open Letter written in accessible language?

Yes

Where applicable, are recommendations and next steps explained clearly for others to follow?

Partly

Is the rationale for the Open Letter provided in sufficient detail?

Yes

Reviewer Expertise:

Climate Science, Climate Services, Climate Risk management

I confirm that I have read this submission and believe that I have an appropriate level of expertise to confirm that it is of an acceptable scientific standard, however I have significant reservations, as outlined above.
